# Natural killer T cells in intracerebral hemorrhage: phase-dependent immune responses and therapeutic implications

**DOI:** 10.3389/fimmu.2026.1824143

**Published:** 2026-05-07

**Authors:** Xu Zhao, Likun Wang, Siying Ren, Zhao Yang, Guofeng Wu

**Affiliations:** 1 Emergency Department, The Affiliated Hospital of Guizhou Medical University, Guiyang, Guizhou, China; 2 Key Lab of Acute Brain Injury and Function Repair in Guizhou Medical University, Guiyang, Guizhou, China; 3 Department of Neurology, The Affiliated Yongchuan Hospital of Chongqing Medical University, Chongqing, China

**Keywords:** blood–brain barrier, immunomodulation, intracerebral hemorrhage, microglia, natural killer T cells, neuroinflammation, precision medicine, α-GalCer

## Abstract

**Background:**

Intracerebral hemorrhage (ICH) is the most lethal subtype of stroke, yet effective disease-modifying therapies remain limited. Beyond the primary mechanical insult, dysregulated neuroinflammation is thought to be a major contributor to hematoma expansion, perihematomal edema, and secondary neuronal injury. Natural killer T (NKT) cells, a specialized population of lipid-reactive lymphocytes linking innate and adaptive immunity, have emerged as potentially relevant immunoregulatory contributors to the post-hemorrhagic response.

**Main body:**

This review summarizes current experimental evidence, together with the still limited clinical data, regarding the biology of NKT cells in ICH, including their development, subset heterogeneity (type I invariant, type II, and regulatory NKT-like programs), and activation through CD1d-restricted lipid antigens and cytokine-driven pathways. Available evidence supports a phase-dependent working model of NKT-cell function in ICH. In the acute stage, type I iNKT-associated responses may amplify neuroinflammation through IFN-γ- and TNF-α-related signaling, thereby contributing to myeloid activation, blood-brain barrier (BBB) dysfunction, and neutrophil recruitment. In later stages, regulatory NKT-associated programs, including type II NKT- or NKT10-like responses, may become increasingly linked to IL-4-, IL-13-, and IL-10-related pathways that support pro-resolving myeloid phenotypes, hematoma clearance, and tissue repair. In addition, NKT cells may influence broader immune networks, including T-cell responses and, more speculatively, B-cell-associated pathways. We also discuss emerging therapeutic strategies targeting NKT-cell biology, including α-GalCer-based ligands, anti-CD1d/TCR approaches, sphingosine-1-phosphate (S1P) receptor modulators, and PPAR-γ agonists, while emphasizing the importance of treatment timing, delivery, and subset specificity.

**Conclusion:**

Current evidence suggests that NKT cells may exert both detrimental and beneficial effects during ICH progression, with their impact varying according to disease stage and microenvironmental context. Their rapid responsiveness and functional plasticity make them promising, though still incompletely validated, targets for phase-tailored immunomodulation. Future progress will depend on high-resolution profiling to better define therapeutic windows, clarify subset-specific functions, and support the development of interventions that more precisely balance inflammatory control with tissue repair.

## 1 Background

Intracerebral hemorrhage (ICH) accounts for approximately 15–20% of all stroke cases and remains the most lethal major stroke subtype, with 30-day mortality rates ranging from 35% to 50% (1, 2). Despite advances in acute care, effective disease-modifying treatments remain limited, and a substantial proportion of survivors are left with persistent neurological deficits (3–5). The pathophysiology of ICH is driven not only by the immediate mechanical effects of hematoma formation but also by a cascade of secondary injury processes shaped by complex neuroinflammatory and immune responses (3, 4, 6–8). Increasing evidence suggests that dysregulated neuroinflammation contributes to hematoma expansion, perihematomal edema, and delayed neuronal death, thereby influencing functional outcomes (3, 5, 6, 8–12). Early studies of post-hemorrhagic neuroinflammation focused predominantly on innate immune responders, such as microglia, astrocytes, and infiltrating macrophages, because of their rapid responses to blood components and damage-associated molecular patterns (DAMPs) (3, 5, 8, 10, 13, 14).

More recent work, however, indicates that adaptive immune cells, including multiple lymphocyte subsets, also shape the inflammatory milieu and may influence both tissue injury and repair (15–20). Accordingly, the current view of ICH has expanded from a predominantly innate-driven process to a broader framework involving dynamic crosstalk between innate and adaptive immunity (4, 5, 8, 15, 19, 21).

Bridging these systems, natural killer T (NKT) cells have emerged as a distinctive lymphocyte subset with features of both natural killer (NK) cells and conventional T lymphocytes (22–24). Unlike classical T cells, which recognize peptide antigens presented by major histocompatibility complex (MHC) molecules, NKT cells respond to lipid antigens presented by the non-polymorphic CD1d molecule (22, 25–28). Once activated, they can rapidly produce both pro- and anti-inflammatory cytokines, thereby influencing local and systemic immune responses (23, 24, 29–33). Given their reported roles in ischemic stroke, subarachnoid hemorrhage, and traumatic brain injury (29, 34–38), NKT cells have emerged as plausible contributors to the pathophysiology of ICH.

However, the specific contribution of NKT cells to ICH remains incompletely defined. Available evidence suggests that NKT-cell responses may be context dependent, with potentially divergent roles in pro-inflammatory injury and immunoregulatory repair (4, 11, 15, 16, 18, 19, 29, 36, 39, 40). This broader concept has also been discussed in a recent Research Highlight on specialized NK T-cell responses in acute brain injury (41). This complexity highlights the need for a more nuanced understanding of their temporal dynamics and subset specificity before therapeutic strategies can be rationally designed. Therefore, this review synthesizes current knowledge of NKT-cell biology in the context of ICH and evaluates their potential as phase-specific therapeutic targets. Specifically, we address three questions: (1) how do the developmental and phenotypic features of NKT cells shape their response to hemorrhage; (2) what are the spatiotemporal patterns of NKT-cell involvement during secondary brain injury after ICH; and (3) how might the dual nature of NKT cells be leveraged through precision immunomodulation to improve outcomes? To support this narrative review, we performed a structured literature search in PubMed (last updated on October 1, 2025). Search terms included combinations of “intracerebral hemorrhage, ” “natural killer T cells, ” “NKT, ” “iNKT, ” “neuroinflammation, ” and “immune regulation.” Reference lists of relevant original articles and reviews were also screened manually to identify additional studies. The present review narratively integrates these experimental and clinical findings, with particular emphasis on temporal dynamics, subset-specific effects, and the therapeutic implications of NKT cells in ICH.

## 2 Biological characteristics of NKT cells

### 2.1 Classification and subgroups

**Figure 1:**
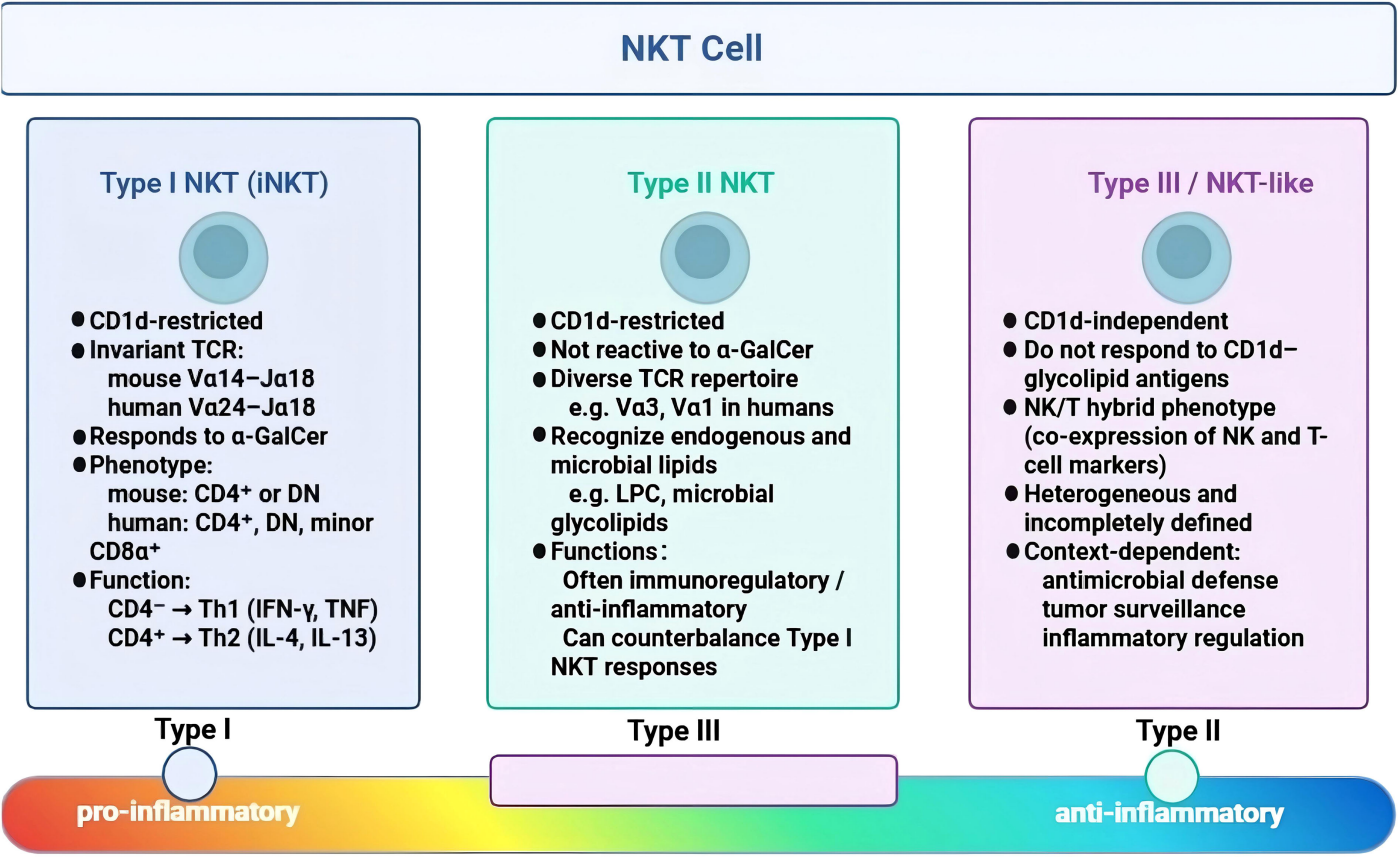
Classification and inflammatory spectrum of NKT-cell subsets. Classical CD1d-restricted NKT cells comprise type I (invariant NKT, iNKT) and type II subsets. A heterogeneous CD1d-independent population is historically termed type III (NKT-like) cells. type I iNKT cells express a semi-invariant TCR and respond to CD1d-presented glycolipids such as α-GalCer, with cytokine profiles ranging from Th1- to Th2-like depending on CD4 expression. type II NKT cells are CD1d-restricted but α-GalCer–unresponsive, exhibit a diverse TCR repertoire, and are often associated with immunoregulatory/anti-inflammatory functions. type III (NKT-like) cells are CD1d-independent, display NK/T hybrid features, and remain incompletely defined. These subsets can be conceptualized as occupying different positions along an inflammatory–regulatory spectrum, although their boundaries and functional states are context dependent.

Natural killer T (NKT) cells are a distinct lineage of unconventional T lymphocytes that bridge innate and adaptive immunity. Characterized by rapid effector responses and the ability to recognize lipid antigens, they occupy an important position in immune surveillance and immunoregulation (22–26, 42, 43). NKT cells are most commonly divided into two classical CD1d-restricted subsets, namely type I and type II NKT cells. In addition, a heterogeneous population historically termed “type III” or “NKT-like” cells has also been described, although these cells are CD1d-independent and are not generally considered classical NKT cells (23, 24, 44–47) (**Figure 1**).

Type I NKT cells, commonly referred to as invariant NKT (iNKT) cells, represent the prototypical CD1d-restricted subset. They express a semi-invariant TCR alpha chain (Vα14-Jα18 in mice and the homologous Vα24-Jα18 in humans) paired with a limited repertoire of beta chains (23, 24, 44, 45, 47, 48). A defining feature of iNKT cells is their recognition of the prototypical glycolipid antigen α-galactosylceramide (α-GalCer) presented by CD1d. Phenotypically, murine iNKT cells are primarily CD4+ or double negative (DN; CD4−CD8−), whereas human iNKT-cell populations include CD4+, DN, and a minor CD4−CD8α+ subset. This surface phenotype is associated with, but does not fully determine, functional polarization: CD4− subsets tend to show a more Th1-skewed profile, whereas CD4+ cells are more often associated with Th2- or regulatory-type cytokine production, including IL-4, IL-13, and in some contexts IL-10 (23, 24, 47, 49, 50).

Type II NKT cells share the CD1d-restriction of iNKT cells but do not recognize α-GalCer. Instead, they utilize a more diverse TCR repertoire (incorporating Vα3, Vα8, or Vα1 elements in mice) to recognize a broad spectrum of endogenous lipid antigens—such as sulfatides and lysophosphatidylcholine (LPC)—as well as microbial glycolipids (24, 30, 46, 51, 52). Functionally, type II NKT cells are frequently associated with immunosuppressive or regulatory roles and have been shown to counterbalance the pro-inflammatory activities of type I NKT cells in various autoimmune and inflammatory models, thereby contributing to the maintenance of immune homeostasis (24, 30, 46, 51, 52).

Type III NKT cells, often termed “NKT-like” cells, represent a heterogeneous group of CD1d-independent T cells that co-express NK cell markers (e.g., NK1.1 in mice or CD56 in humans). Unlike classical NKT cells, they recognize peptide antigens via conventional MHC molecules yet retain innate-like effector functions. Although this subset remains less characterized in the context of stroke, accumulating evidence suggests that NKT-like cells actively participate in cytotoxic defense, tumor surveillance, and inflammatory modulation under specific pathological conditions (23, 24, 42, 43, 53).

Collectively, these NKT-cell subsets and NKT-like populations represent a versatile immunoregulatory compartment, capable of driving dichotomous outcomes—from potent inflammation to resolution—depending on the cytokine milieu, lipid antigen availability, and tissue microenvironment (23, 24, 30, 32, 42–47). In the complex pathology of ICH, this functional heterogeneity implies that distinct NKT-cell subsets likely exert divergent, and potentially opposing, effects on neuroinflammation and tissue repair across different stages of the disease progression.

### 2.2 Development and tissue distribution of NKT cells

NKT cells follow a unique developmental trajectory in the thymus, distinct from conventional T cells, which endows them with innate-like functional readiness even before antigen encounter. During thymic selection, NKT precursors undergo a specialized differentiation program characterized by pre-formed transcripts for cytokines such as IFN-γ and IL-4 (23, 44, 54). This distinct ontogeny implies that genetic polymorphisms or environmental factors influencing thymic NKT maturation could set the threshold for their activation following acute cerebral insults like ICH (55).

Upon egress from the thymus, NKT cells exhibit a striking tissue-specific distribution pattern. In mice, invariant NKT (iNKT) cells are disproportionately enriched in the liver, constituting approximately 20–30% of hepatic T lymphocytes, and are also abundant in the spleen and bone marrow. In contrast, human iNKT cells are comparatively rare in peripheral blood (0.01–1% of T cells) but show significant enrichment in specific tissues, including the liver and omentum, where they retain features of tissue residency and rapid responsiveness (23, 24, 47, 49, 54, 56).Despite these quantitative differences across species, the ability of NKT cells to function as early responders appears to be evolutionarily conserved, supporting their critical role in local and systemic immune surveillance.

This preferential hepatic residency places iNKT cells in a position to respond rapidly to sterile injury signals or microbial products through cytokine secretion and interaction with Kupffer cells, hepatocytes, and other immune populations (49, 56–59). In the context of ICH, this systemic reservoir raises the possibility that hepatic and splenic NKT cells may contribute to early brain–periphery immune communication after hemorrhage. However, the specific signals governing their mobilization and functional effects in ICH remain incompletely defined.

### 2.3 Activation mechanisms

**Figure 2:**
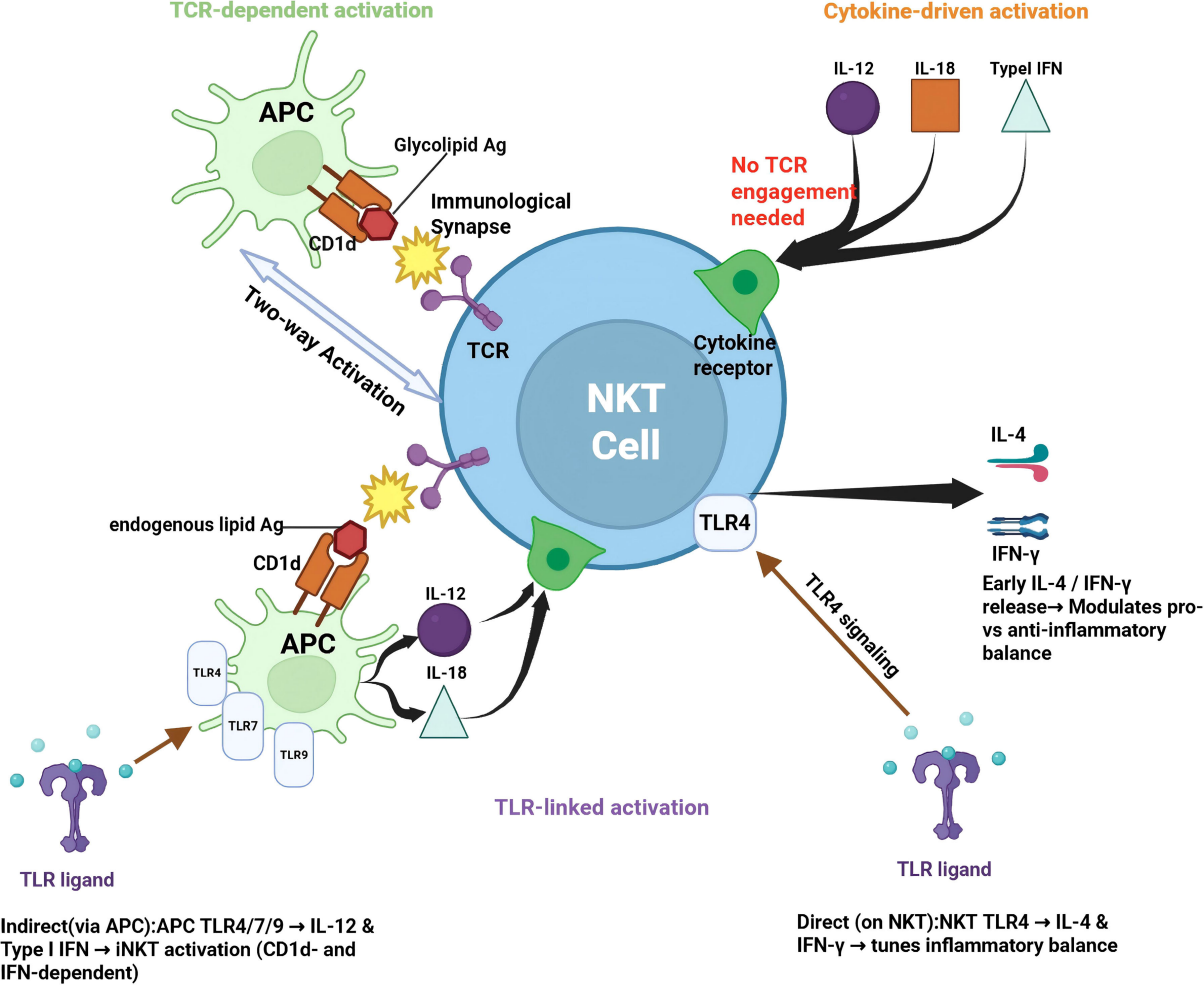
Principal mechanisms of NKT-cell activation. NKT cells can be activated through three major pathways reflecting their innate–adaptive immune properties. TCR-dependent activation is initiated by recognition of CD1d-presented glycolipid antigens (e.g., α-GalCer) on APCs, resulting in rapid bidirectional activation. Cytokine-driven activation occurs independently of TCR engagement and is mediated by inflammatory cytokines such as IL-12, IL-18, and type I interferons, particularly in acute inflammatory settings. TLR-linked activation involves indirect stimulation via TLR signaling in APCs that enhances cytokine production and endogenous lipid presentation, as well as direct TLR signaling in NKT cells. These activation routes converge to induce rapid cytokine production and may enable NKT cells to influence inflammatory responses after ICH, although the relative contribution of each pathway likely varies with context and disease stage.

NKT cells can be activated through three distinct but interconnected pathways, reflecting their unique position at the innate–adaptive interface (**Figure 2**).

#### 2.3.1 TCR-dependent activation

The canonical activation pathway is triggered by the recognition of lipid antigens presented by the CD1d molecule on antigen-presenting cells (APCs), such as dendritic cells, macrophages, and potentially microglia. In type I iNKT cells, this interaction relies on engagement of the semi-invariant TCR by glycolipid–CD1d complexes, forming a specialized immunological synapse that facilitates rapid signaling (22, 23, 25–28). Notably, this interaction is bidirectional: while the TCR signal activates the NKT cell to release cytokines, the ligation of CD40 on the APC by CD40L on the NKT cell further amplifies APC maturation and IL-12 production. Structural studies have shown that the geometry of lipid antigens within the CD1d-binding groove, together with the mode of TCR engagement, strongly influences the quality and magnitude of iNKT-cell activation, with prototypical ligands such as α-GalCer eliciting robust responses (27, 48). In the context of sterile inflammation like ICH, endogenous lipid antigens released from damaged cell membranes are likely to contribute to this pathway, although the dominant lipid species and their relative importance in ICH remain incompletely defined.

#### 2.3.2 Cytokine-driven activation

Even in the absence of direct TCR engagement, NKT cells can be rapidly activated by a cytokine-rich inflammatory milieu. Pro-inflammatory cytokines, particularly IL-12, IL-18, and type I interferons, can act synergistically to induce NKT cells to produce substantial amounts of IFN-γ (23, 32, 33, 60, 61). This “cytokine-first” or “bystander” activation mode is especially relevant in the hyperacute phase of brain injury (e.g., within minutes to hours of hemorrhage), where a surge of cytokines may precede efficient antigen processing and presentation (4, 5, 9, 29, 34, 62). This mechanism allows NKT cells to amplify the early inflammatory response even before specific lipid antigens become available.

#### 2.3.3 TLR-linked activation

Toll-like receptors (TLRs) orchestrate NKT cell activation through both indirect and direct mechanisms. Indirectly, TLR engagement on APCs (e.g., TLR4, TLR7, and TLR9) enhances the expression of CD1d and co-stimulatory molecules while stimulating the production of IL-12 and type I IFNs, thereby lowering the threshold for NKT activation (33, 61, 63). Furthermore, direct TLR signaling on NKT cells themselves has been documented. For instance, direct TLR4 stimulation on activated iNKT cells can modulate their cytokine profile, skewing the balance between early IL-4 and IFN-γ secretion (23, 33, 50, 61, 64). This TLR-mediated pathway provides a mechanism for NKT cells to sense danger signals (DAMPs) released from the hematoma and necrotic brain tissue.

Regardless of the initial trigger, activated NKT cells rapidly secrete a diverse array of Th1-associated (e.g., IFN-γ, TNF-α) and Th2-/regulatory-associated (e.g., IL-4, IL-10, IL-13) cytokines, often within hours. Through these immediate effector functions and the upregulation of surface molecules such as CD40L, NKT cells can shape downstream innate and adaptive immune responses (23, 24, 29, 32, 33, 45, 47, 58, 65, 66). Understanding the temporal interplay among these activation pathways after ICH may help identify time-resolved therapeutic strategies. For example, limiting early cytokine-driven inflammatory amplification while preserving potentially beneficial later immune programs may prove more effective than indiscriminate NKT suppression (4, 8, 11, 15, 29, 34, 36, 40).

## 3 Dynamic changes of NKT cells after ICH

### 3.1 Temporal dynamic characteristics

Following ICH, available evidence suggests that NKT-cell responses may evolve in a phase-dependent manner broadly paralleling the temporal progression of secondary brain injury (4, 8, 11, 15, 18, 29, 34, 36, 39, 40). In the early phase, NKT-associated responses are thought to be predominantly pro-inflammatory and may contribute to amplification of innate neuroinflammation and blood-brain barrier (BBB) disruption. In later phases, these responses may become increasingly associated with anti-inflammatory and reparative programs that could facilitate hematoma clearance and tissue remodeling (4, 5, 9, 29, 34–36, 67–71). Thus, rather than being uniformly pathogenic or protective, NKT cells are better viewed as context-dependent and potentially phase-specific immunoregulators. One possible interpretation is that such a biphasic pattern reflects a broader immune program that may be adaptive in infectious settings, where early inflammatory activation is followed by regulatory mechanisms that limit collateral damage. In sterile brain injury such as ICH, however, this same program may become maladaptive: excessive early inflammation may aggravate tissue injury, whereas later reparative responses may be insufficient, delayed, or spatially restricted, thereby compromising long-term neurological recovery (3, 5, 7–9, 62, 72–75).

#### 3.1.1 Early phase (approximately 1–3 days)

In the acute phase of ICH, danger-associated molecular patterns (DAMPs) and early inflammatory cytokines, notably IL-12 and IL-18, are proposed to promote rapid activation of NKT cells and may facilitate redistribution of these cells from peripheral reservoirs such as the liver and spleen (23, 24, 33, 49, 56, 60, 62, 76). DAMP-induced signaling through pattern-recognition receptors (PRRs) may also prime antigen-presenting cells to present endogenous lipids on CD1d, thereby further supporting iNKT-cell activation (22, 23, 60, 62, 76). This early response has been associated with upregulation of activation markers such as CD69 and CD25 and with a Th1-skewed cytokine profile, including IFN-γ and TNF-α, although direct NKT-resolved evidence in ICH remains limited. Such responses are broadly consistent with enhanced leukocyte recruitment, microglial activation, and intensified perihematomal neuroinflammation (4, 9, 62, 72, 74, 76, 77).

Mechanistically, while this early wave is likely intended to mobilize host defense rapidly, in the sterile context of a hematoma it may also aggravate BBB breakdown, vasogenic edema, and neuronal death (3, 5, 7, 9, 62, 72–76). Accordingly, early NKT-cell activation in ICH may represent a double-edged response, combining immune surveillance functions with the potential to exacerbate secondary injury.

#### 3.1.2 Late phase (approximately 3–7 days)

**Figure 3:**
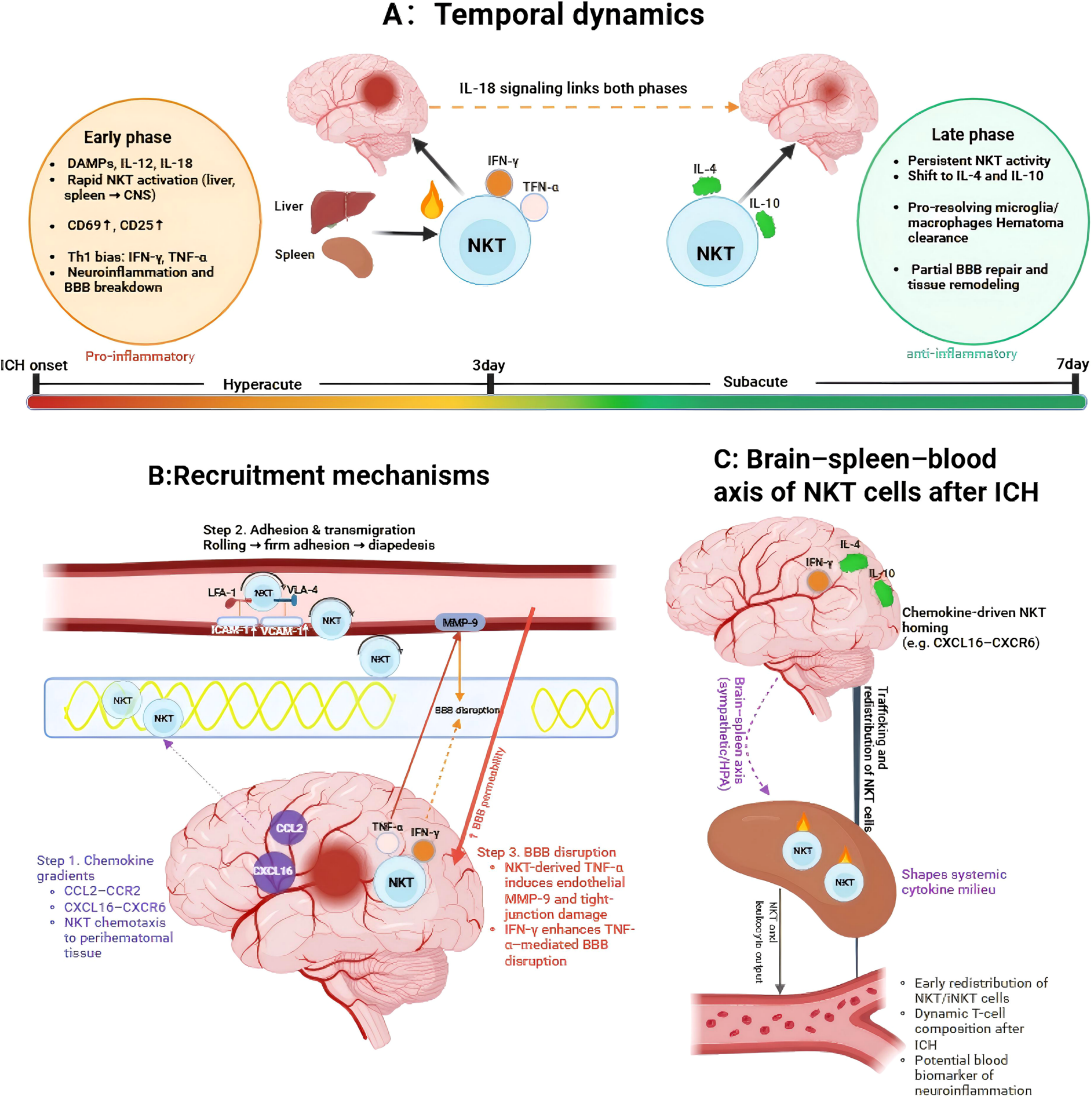
Spatiotemporal dynamics and systemic recruitment of NKT cells following intracerebral hemorrhage. **(A)** Temporal dynamics: Upon ICH onset, DAMPs and early cytokines such as IL-12 and IL-18 are proposed to promote rapid NKT-cell activation in peripheral reservoirs, including the liver and spleen. In the hyperacute/early phase (<3 days), NKT-associated responses may show a predominantly pro-inflammatory, Th1-skewed profile, characterized by activation markers such as CD69 and CD25 and production of IFN-γ and TNF-α, potentially contributing to neuroinflammation and BBB disruption. By the subacute/late phase (3–7 days), these responses may become increasingly associated with anti-inflammatory or reparative programs, including IL-4- and IL-10-related pathways, that could support hematoma clearance, microglial resolution, and tissue remodeling. IL-18 signaling may contribute across phases. **(B)** Recruitment mechanisms: NKT-cell recruitment is hypothesized to involve several coordinated steps, including: (1) chemotaxis along gradients involving the CCL2-CCR2 and CXCL16-CXCR6 axes toward the perihematomal region; (2) adhesion and diapedesis mediated by interactions such as LFA-1/ICAM-1 and VLA-4/VCAM-1 at activated endothelium; and (3) BBB modulation, in which NKT-derived cytokines may contribute to endothelial activation, MMP-9 induction, and tight-junction disruption, thereby facilitating additional immune-cell entry. **(C)** Brain-spleen-blood axis: The figure illustrates bidirectional communication between the injured brain and peripheral immune organs. Sympathetic nervous system (SNS) and hypothalamic-pituitary-adrenal (HPA) axis signals may contribute to splenic immune-cell redistribution, alter the systemic cytokine milieu, and shape circulating immune composition, with possible implications for blood-based biomarkers of neuroinflammation severity.

As injury evolves into the subacute phase, NKT-cell activity may persist but become increasingly associated with anti-inflammatory and reparative programs. During this period, increased IL-4- and IL-10-associated signaling, together with induction of tissue-repair pathways, has been linked in experimental systems to attenuation of neuroinflammation, hematoma resolution, and early recovery processes (4, 5, 29, 34–36, 68, 69, 71, 78, 79). Persistent IL-18 signaling may help sustain NKT-cell engagement across phases, although whether it serves as a functional bridge between inflammatory and reparative programs in ICH remains incompletely defined (33, 60, 62). Functionally, this later phase may involve support for microglial/macrophage polarization toward phagocytic, M2-like, or other pro-resolving states, thereby promoting debris clearance and partial restoration of BBB integrity (4, 5, 68, 69, 71, 80–82). In this sense, NKT cells may contribute to the transition from destructive inflammation toward resolution. From a therapeutic perspective, this working model suggests that time-resolved modulation of NKT-cell responses may be more informative than indiscriminate suppression. In principle, selectively enhancing regulatory NKT programs, including NKT10-like responses, could support endogenous repair, although this remains speculative in ICH. Collectively, these observations support a working model in which NKT cells may act as dynamic temporal regulators after ICH, contributing to both early inflammatory amplification and later immune resolution. A clearer understanding of this timeline will be important for therapeutic design, because modulation at the wrong phase could be ineffective or even harmful (see **Figure 3** for a schematic overview).

### 3.2 Recruitment mechanisms

Recruitment of NKT cells to sites of ICH is likely to involve a multistep process shaped by chemotactic gradients, adhesion dynamics, and BBB dysfunction, although much of the supporting evidence remains indirect or extrapolated from broader neuroinflammatory settings (4, 7–9, 72, 73, 83, 84) (**Figure 3**).

#### 3.2.1 Chemokine gradients

Following ICH, injured neural tissue releases a complex chemokine milieu that directs immune-cell migration. Among these signals, CCL2, produced primarily by activated microglia and macrophages, is likely to contribute to early immune-cell recruitment into the perihematomal region and may also influence NKT-cell trafficking through CCR2, although the specific importance of the CCL2-CCR2 axis for NKT cells in ICH remains to be established (8, 10, 13, 73, 84–86). In parallel, the CXCL16-CXCR6 axis represents a plausible pathway for recruitment, retention, and activation of CXCR6+ NKT cells in inflamed CNS tissue, based on evidence from other neuroinflammatory settings and emerging data relevant to CNS immune trafficking (18, 87–89). Together, these chemotactic cues may help shape local immune-cell positioning around necrotic and gliotic regions during the acute inflammatory phase.

#### 3.2.2 Adhesion molecule–mediated rolling and extravasation

Chemokine-induced integrin activation is likely to promote interactions between LFA-1 (αLβ2) and ICAM-1, as well as between VLA-4 (α4β1) and VCAM-1, both of which are upregulated on cerebral endothelium after ICH (5, 9, 72, 90). These ligand-receptor interactions facilitate firm adhesion and transendothelial migration of infiltrating immune cells and may similarly support NKT-cell entry into the brain parenchyma. Clinical and experimental studies have documented early ICAM-1 and VCAM-1 expression within the first 3 days after hemorrhage and have shown associations with immune infiltration, secondary tissue injury, and poorer neurological outcomes (5, 90). Thus, the adhesion cascade may influence not only overall leukocyte trafficking but also the degree to which NKT-cell recruitment contributes to secondary injury.

#### 3.2.3 Blood–brain barrier disruption

Mechanical injury and inflammatory signaling act synergistically to compromise the BBB after ICH. NKT-derived cytokines such as TNF-α and IFN-γ could plausibly contribute to endothelial activation, MMP-9 induction, and tight-junction disruption, although the specific quantitative contribution of NKT cells relative to other immune sources remains unclear (23, 24, 91–93). In this framework, cytokine-MMP interactions may reinforce BBB permeability, facilitate subsequent immune-cell infiltration, and amplify neuroinflammation within the perihematomal microenvironment (4, 5, 8, 9, 72, 75, 91–93). Taken together, these observations suggest that NKT-cell recruitment after ICH is unlikely to be merely a passive consequence of barrier failure. Rather, it is more plausibly shaped by coordinated interactions among chemotactic signaling, adhesion cascades, and BBB plasticity (4, 7–9, 72, 73, 84, 90). These processes may offer multiple candidate intervention points, including chemokine receptors, integrins, and MMP-related pathways, for modulating NKT-cell trafficking and function in ICH.

### 3.3 Tissue-specific reactions

#### 3.3.1 Peripheral blood

During the hyperacute to early subacute phases of ICH, peripheral blood serves as a dynamic conduit for immune-cell mobilization. Circulating unconventional T-cell subsets, including NKT cells, may undergo redistribution as immune cells traffic between the circulation, secondary lymphoid organs, and the injured brain (4, 7, 8, 15, 18, 19, 94). In clinical cohorts, rapid alterations in peripheral T-cell composition correlate with hematoma volume and neurological deterioration, and similar patterns have been described in ischemic stroke immunology (7, 9, 18, 19, 94). In experimental ICH models, single-cell analyses have also identified early activation and distinct trafficking-related programs among innate-like T-cell populations (8, 10, 13, 15, 18, 19, 34). Direct clinical data specifically tracking NKT-cell dynamics in ICH remain sparse. However, studies in ischemic stroke provide a potentially informative translational framework, in which reduced circulating iNKT-cell frequencies have been associated with increased infection risk and poorer functional outcomes (35), possibly reflecting tissue redistribution, activation-induced cell death, or altered systemic immune regulation (29, 34, 35). Given their potent cytokine-producing capacity (22, 23, 29, 32, 33, 42–47, 71, 95).

#### 3.3.2 Spleen

The spleen is likely to function as an important immune reservoir and relay organ after cerebral hemorrhage. The so-called brain-spleen axis is rapidly engaged after ICH, with sympathetic nervous system activation and catecholamine release contributing to splenic contraction and lymphocyte egress (7, 96, 97). This neuroimmune crosstalk is further influenced by the hypothalamic-pituitary-adrenal axis, together shaping a systemic environment that may favor either immunosuppression or inflammatory dysregulation depending on timing and context. Splenic contraction and lymphocyte mobilization after ICH could include redistribution of NKT cells, although direct evidence specifically quantifying splenic NKT release in ICH remains limited (23, 24, 29, 32, 42–47, 93, 95). Once mobilized, these cells may traffic to injured tissues or influence the systemic cytokine milieu through secretion of IFN-γ, IL-4, IL-10, and related mediators. Thus, rather than acting as a passive storage organ, the spleen may serve as an important intermediary linking CNS injury to systemic immune responses that subsequently feedback on brain pathology (4, 7, 8, 15, 29, 34, 36, 72, 96, 97).

#### 3.3.3 Brain

Within the brain parenchyma, immune-cell infiltration follows a defined spatiotemporal course after ICH. In murine models, lymphocytes begin to accumulate in the perihematomal region within approximately 24–72 hours, with broader immune-cell infiltration peaking around days 3–7 (4, 7, 8, 15, 18, 19, 34). Whether NKT cells follow precisely the same kinetics remains incompletely resolved. However, available evidence suggests that at least a subset of NKT-associated responses may emerge within this window as the local inflammatory milieu matures. Early NKT-associated signaling in the brain is hypothesized to include IFN-γ-dominant responses that may promote microglial priming and downstream IL-1β production, thereby contributing to secondary injury (10, 15, 18, 23, 29, 34, 72, 93, 98). In later phases, IL-4-associated NKT programs may support a shift toward M2-like or other pro-resolving myeloid phenotypes, thereby promoting hematoma clearance and tissue remodeling (67, 68, 70, 72, 73). In addition, the CXCL16-CXCR6 axis has emerged as a potentially important pathway for NKT-cell homing and retention within inflamed CNS tissue (87–89), although its precise role in perihematomal NKT localization requires further clarification. Overall, these tissue-specific observations support a unified working model in which NKT-cell redistribution after ICH may be shaped by chemokine gradients, adhesion interactions, and BBB dysfunction across the blood, spleen, and brain. This putative brain-spleen-blood axis links peripheral immune mobilization to local neuroinflammation and may help shape the balance between injury progression and functional recovery after ICH.

## 4 The dual role of NKT cells in the pathophysiology of ICH

**Figure 4:**
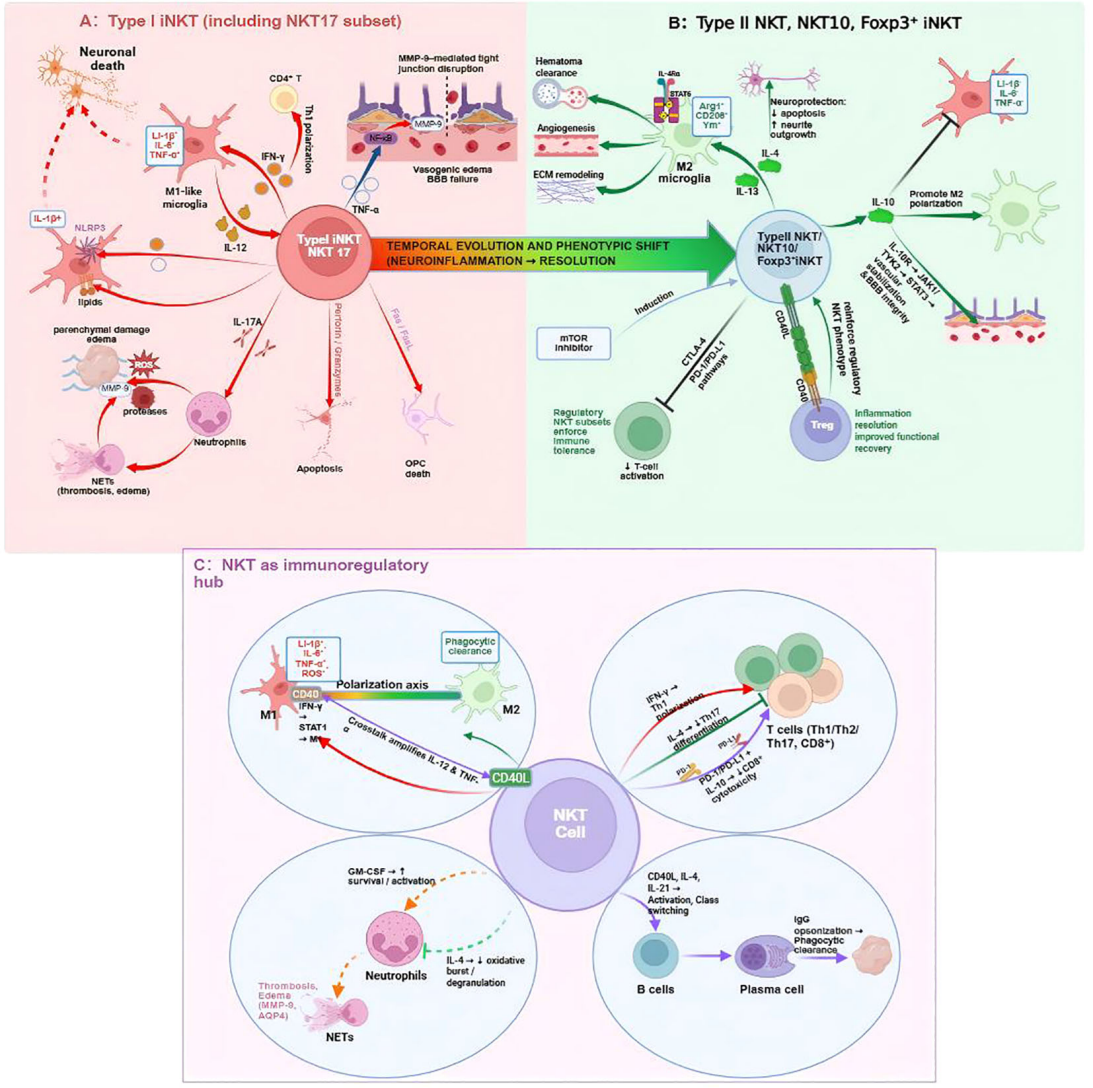
Proposed phase-dependent and pleiotropic functions of NKT cells in ICH pathophysiology. **(A)** Early pro-inflammatory phase: Following hemorrhage, type I iNKT-associated responses may be triggered by endogenous lipid antigens, inflammatory cytokines, and danger-associated molecular patterns (DAMPs). These early responses may involve IFN-γ-, TNF-α-, and IL-17-related pathways and could contribute to microglial activation, neutrophil recruitment, matrix metalloproteinase induction, BBB dysfunction, and secondary neural injury. **(B)** Resolution and repair phase: As the inflammatory milieu evolves, some NKT-cell programs may become increasingly associated with regulatory and reparative functions, including IL-4-, IL-10-, and IL-13-related signaling. These responses may support phagocytic and pro-resolving myeloid phenotypes, hematoma clearance, and neurovascular remodeling. **(C)** Immunoregulatory interactions: Beyond this temporal framework, NKT cells may act as immunoregulatory nodes linking innate and adaptive immunity. Through cytokine secretion and contact-dependent signaling, they may influence neutrophil activation, myeloid polarization, T-cell differentiation, and, more speculatively, B-cell responses. The relative contribution of these interactions to ICH pathophysiology remains incompletely defined.

Available evidence suggests that NKT-cell responses in ICH are functionally heterogeneous and may be phase-dependent, with potentially divergent roles in early injury amplification and later immune resolution. In the hyperacute and acute stages, type I iNKT-associated programs, including NKT1- or NKT17-like responses, may contribute to secondary brain injury by amplifying neuroinflammation and interacting with other innate immune pathways (4, 7–9, 11, 15, 18, 29, 34, 36, 72–75). In later stages, regulatory NKT programs, including type II NKT- or NKT10-like responses, may become increasingly associated with immune resolution, hematoma clearance, and tissue repair (4, 5, 8, 11, 29, 67–71, 78–82, 99, 100). This conceptual framework broadly parallels the temporal dynamics described above and supports the idea that NKT cells may exert both deleterious and beneficial effects depending on disease stage and microenvironmental context (**Figure 4**).

### 4.1 Pro-inflammatory roles

In the immediate aftermath of hemorrhage, NKT-associated responses may participate in initiation or amplification of the neuroinflammatory cascade. Rather than acting through a single pathway, these effects likely reflect several overlapping mechanisms involving cytokine production, cellular crosstalk, and, in some settings, direct cytotoxicity.

#### 4.1.1 IFN-γ production

IFN-γ-related signaling is one of the most plausible mechanisms by which NKT cells may enhance early inflammatory injury. In experimental systems, IFN-γ produced by NKT1-like cells can promote microglial activation, increase antigen-presenting capacity, and enhance release of pro-inflammatory mediators such as IL-1β, IL-6, and nitric oxide (4, 5, 72, 98, 101). This may create a feed-forward loop in which activated myeloid cells produce IL-12, thereby further supporting NKT-cell activation and Th1-skewed responses (23, 29, 34, 72, 98, 102). Although direct evidence specifically isolating NKT-derived IFN-γ in ICH remains limited, available data support the view that IFN-γ-associated NKT responses may contribute to early perihematomal inflammation and edema formation (4, 7–9, 15, 62, 72–74, 98, 101).

#### 4.1.2 TNF-α secretion and BBB disruption

TNF-α-related signaling provides another plausible route by which activated NKT cells may aggravate secondary injury. TNF-α can impair endothelial integrity by downregulating tight-junction proteins and by promoting MMP-9 expression in cerebral microvessels (4, 5, 9, 72–74, 91–93, 102). In principle, NKT-derived TNF-α could therefore contribute to BBB dysfunction, vasogenic edema, and downstream neuronal or oligodendroglial stress. However, the quantitative contribution of NKT cells relative to other immune and stromal sources of TNF-α in ICH remains unclear. Thus, TNF-α-related NKT responses are best viewed as a plausible component of the broader inflammatory network that drives edema formation in the acute stage.

#### 4.1.3 IL-17-associated responses

A subset of iNKT cells can adopt an NKT17-like phenotype and produce IL-17A (23, 24, 50, 87, 103). In inflammatory settings, IL-17 is known to promote neutrophil-recruiting chemokine programs, including CXCL1 and CXCL2, and may thereby indirectly enhance neutrophil infiltration (23, 24, 50, 87, 103). In ICH, this mechanism is biologically plausible because neutrophils contribute to oxidative stress, protease release, and tissue edema (4, 5, 8, 72–75, 84). However, direct evidence establishing NKT17 cells as major drivers of neutrophil-mediated injury in ICH remains limited. Accordingly, IL-17-associated NKT responses should currently be interpreted as a candidate mechanism rather than a fully established pathway in ICH pathogenesis.

#### 4.1.4 Cytotoxic effector pathways

Activated iNKT cells possess cytolytic machinery, including perforin/granzyme and Fas/FasL pathways (15, 18, 23, 24, 29, 34, 104–106). These pathways could, in principle, contribute to direct injury of stressed neurons or vulnerable glial populations. Some experimental observations in related settings suggest that NKT cells can display enhanced cytotoxicity under inflammatory conditions (15, 18, 29, 34, 35, 106). Nevertheless, the extent to which direct contact-dependent NKT cytotoxicity contributes meaningfully to tissue damage after ICH remains incompletely defined. At present, this mechanism should be considered plausible but not yet well resolved *in vivo*.

#### 4.1.5 Crosstalk with inflammasome pathways

NKT-associated cytokine signaling may also interact with inflammasome pathways, including NLRP3-related responses, in microglia and infiltrating macrophages (4, 8, 9, 18, 72, 74, 75). For example, IFN-γ-, TNF-α-, and IL-18-related signaling could contribute to an inflammatory milieu that favors IL-1β maturation and pyroptotic injury. This raises the possibility of a feed-forward loop in which innate inflammasome activation and NKT-cell activation reinforce one another. However, direct mechanistic evidence defining a discrete NKT–NLRP3 axis in ICH remains limited. It is therefore more accurate to describe inflammasome crosstalk as a potentially important amplification mechanism rather than a conclusively established pathway.

Taken together, these observations suggest that early NKT-associated responses may exacerbate secondary brain injury through a combination of IFN-γ- and TNF-α-related inflammation, possible IL-17-driven neutrophil recruitment, interactions with myeloid inflammasome pathways, and, perhaps to a lesser extent, direct cytotoxicity. The relative contribution of each mechanism likely varies across models, time points, and tissue compartments.

### 4.2 Anti-inflammatory and protective effects

In later phases of ICH, or under specific regulatory conditions, some NKT-cell programs may become increasingly associated with immune resolution and repair. These later responses are hypothesized to include anti-inflammatory cytokine production, support for pro-resolving myeloid phenotypes, and interactions with regulatory lymphocyte populations. However, many of these mechanisms remain incompletely defined in ICH and are informed in part by findings from broader NKT biology and related CNS injury models.

#### 4.2.1 IL-4- and IL-13-associated programs

Type II NKT cells and NKT2-like programs are recognized sources of IL-4 and IL-13 (24, 30, 67–71, 79, 107). Through IL-4Rα-STAT6 signaling, these cytokines may promote microglial and macrophage polarization toward phagocytic, M2-like, or other pro-resolving states characterized by markers such as Arg1 and CD206 (24, 30, 67–71, 79, 107). In ICH, such a shift could support hematoma clearance, extracellular matrix remodeling, and tissue repair (68–70, 108, 109). In addition, IL-4- and IL-13-associated signaling may exert indirect neuroprotective effects by limiting inflammatory damage and favoring a reparative microenvironment. While this therapeutic rationale is biologically attractive, direct evidence specifically demonstrating that IL-4/IL-13-producing NKT subsets drive recovery in ICH remains limited.

#### 4.2.2 IL-10 and resolution of neuroinflammation

IL-10-producing NKT programs, including NKT10-like responses, are plausible contributors to inflammatory resolution (24, 30, 32, 109–111). IL-10 signaling suppresses production of pro-inflammatory mediators such as TNF-α, IL-1β, and IL-6 and can limit inflammasome activity in neighboring glial and myeloid cells (79, 110, 112–114). In this framework, NKT-associated IL-10 production may help preserve BBB integrity and reduce persistence of neuroinflammation. Clinical studies showing that higher IL-10 levels in perihematomal tissue or cerebrospinal fluid are associated with reduced edema or better outcomes support the relevance of IL-10 biology in ICH (115), although these observations are not NKT specific. Thus, IL-10-related NKT responses remain a promising but incompletely validated component of the reparative phase.

#### 4.2.3 Regulatory NKT subsets and checkpoint pathways

Emerging studies have identified regulatory NKT populations, including IL-10-producing NKT10-like cells and, in some settings, Foxp3+ NKT-like populations, with immunosuppressive properties (32, 78, 99, 116, 117). These cells may act through soluble mediators such as IL-10 and TGF-β as well as through checkpoint-related pathways including CTLA-4 and PD-1/PD-L1 (78, 99, 102, 104, 116, 117). In principle, such mechanisms could dampen excessive effector responses and promote immune tolerance after brain injury. However, the ontogeny, trafficking, and functional relevance of these regulatory NKT populations in ICH remain insufficiently characterized. Their translational importance should therefore be presented as an emerging concept rather than an established feature of ICH biology.

#### 4.2.4 Synergy with Tregs and immune tolerance circuits

NKT cells may also engage in reciprocal crosstalk with conventional regulatory T cells (Tregs) (32, 78, 79, 116, 118–120). Experimental studies in immunological and CNS injury settings suggest that this interaction can involve CD40-CD40L signaling and cytokine exchange, including IL-2 and IL-10, and may enhance regulatory immune tone. This raises the possibility that coordinated NKT-Treg responses could support inflammatory resolution and tissue repair after ICH. However, direct evidence specifically defining the NKT-Treg axis in ICH remains limited, and much of the current rationale is extrapolated from related disease models. Accordingly, this pathway is best framed as a plausible regulatory circuit that warrants targeted investigation.

Overall, these findings support the view that NKT cells may contribute not only to early inflammatory injury but also to later immune resolution. IL-4-, IL-13-, and IL-10-associated programs, regulatory NKT-like subsets, and interactions with Tregs may all participate in this shift, but the relative importance of each mechanism in ICH remains to be clarified.

### 4.3 Regulation of other immune cells

Beyond their direct effector functions, NKT cells may act as immunoregulatory nodes linking innate and adaptive immune responses after ICH. Through cytokine secretion and cell-cell interactions, they may influence neutrophils, microglia/macrophages, T cells, and, more speculatively, B cells.

#### 4.3.1 Impact on neutrophils

NKT cells may modulate neutrophil recruitment and activation through cytokines such as IL-17, GM-CSF, IL-4, and IL-10 (10, 23, 24, 32, 50, 71, 72, 99). In early inflammatory phases, IL-17- or GM-CSF-associated signals may favor neutrophil survival, trafficking, and effector activation, thereby contributing to oxidative stress, protease release, and possibly NET formation (4, 5, 72–75). During later stages, anti-inflammatory NKT-associated cytokines such as IL-4 and IL-10 may help restrain excessive neutrophil activation and limit collateral injury. Thus, NKT cells may influence neutrophil biology in a bidirectional manner, although the magnitude of this effect in ICH remains incompletely resolved.

#### 4.3.2 Impact on microglia and macrophages

Among the downstream targets of NKT-cell signaling, microglia and infiltrating macrophages are likely to be particularly important. IFN-γ-dominant responses may favor pro-inflammatory, M1-like activation states, whereas IL-4/IL-13-associated responses may promote phagocytic and reparative myeloid phenotypes (24, 30, 67, 71, 79, 80, 107–109). This interaction is also potentially bidirectional, because activated myeloid cells can present lipid antigens via CD1d and produce cytokines such as IL-12 that further shape NKT-cell responses. In this sense, NKT cells and myeloid cells may form a dynamic feedback loop that helps determine whether perihematomal inflammation remains destructive or transitions toward resolution.

#### 4.3.3 Impact on T cells

NKT cells may influence the adaptive T-cell compartment through both cytokine-mediated and contact-dependent mechanisms (23, 24, 29, 32, 34, 42, 43, 79, 99, 104, 105). Depending on context, NKT-derived cytokines may skew helper T-cell polarization and may also restrain excessive effector T-cell activation through IL-10- or checkpoint-related pathways. In ICH, where dysregulated T-cell responses have been implicated in delayed injury and post-stroke immune dysfunction, such interactions may be biologically important. However, the precise balance by which NKT cells shape Th1, Th2, Th17, CD8+, or regulatory T-cell responses after ICH remains insufficiently defined.

#### 4.3.4 Impact on B cells

Interactions between NKT cells and B cells are well established in other immunological settings, including through CD40L-mediated costimulation and cytokines such as IL-4 and IL-21 (79, 116, 119, 121–124). Follicular helper NKT-like programs have also been described in germinal-center contexts (123–125). By contrast, the relevance of these pathways to ICH remains largely speculative. Although it is conceivable that NKT-B-cell interactions could influence humoral immunity or phagocytic clearance of blood-derived products after hemorrhage, direct evidence in ICH is currently sparse. This area therefore remains an interesting but still underdeveloped aspect of NKT biology in hemorrhagic stroke.

Collectively, these observations suggest that NKT cells may help coordinate the broader immune landscape after ICH rather than acting only as isolated effector cells. By shaping neutrophil behavior, myeloid polarization, adaptive T-cell responses, and possibly humoral immunity, NKT cells may influence the overall trajectory from acute injury toward repair. At present, however, many of these interactions are best understood as components of a working model that requires further validation in ICH-specific systems.

## 5 Therapeutic strategies targeting NKT cells

**Figure 5:**
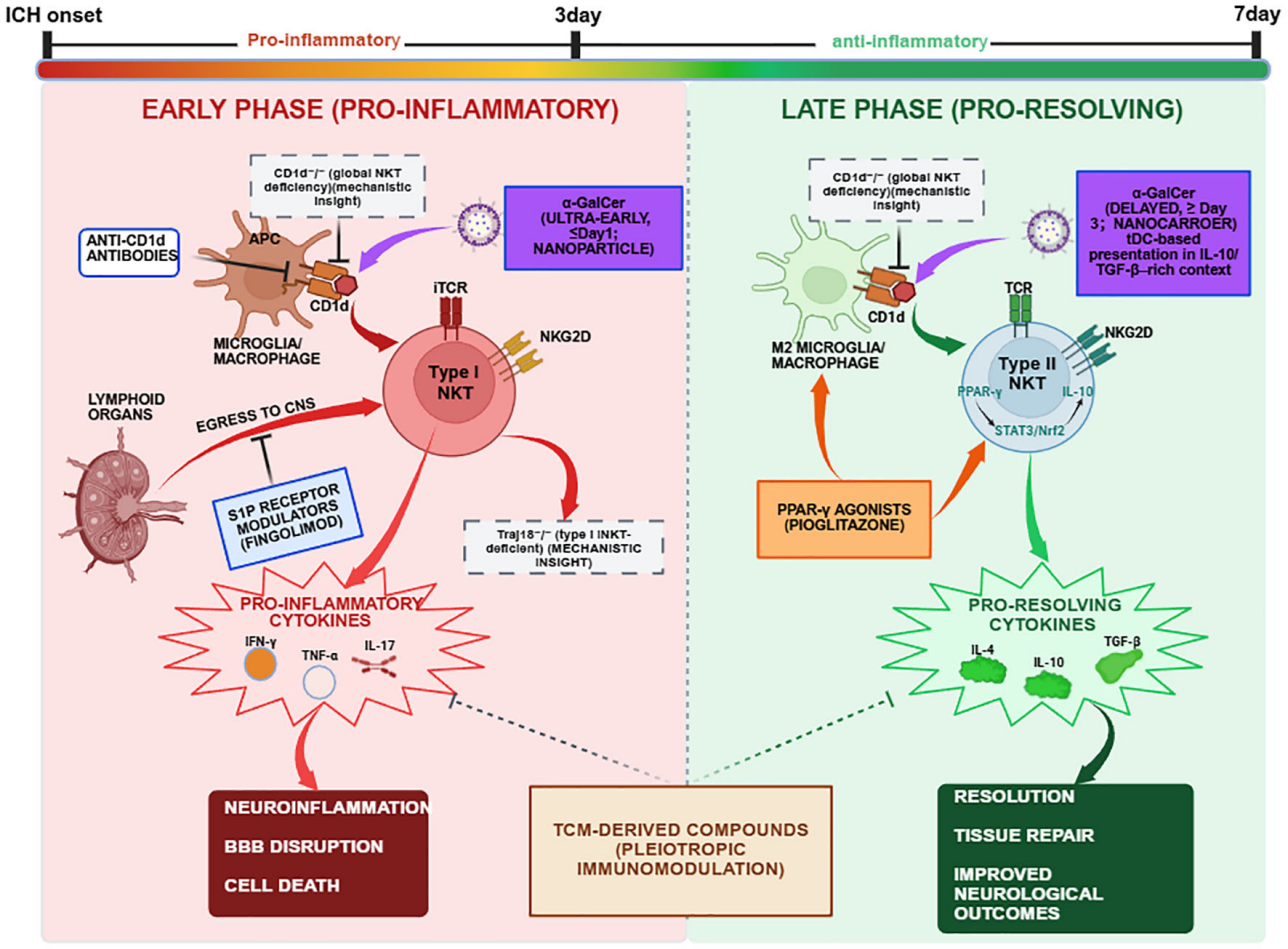
Working model of phase-dependent immune responses and potential therapeutic modulation of NKT cells after ICH. The post-ICH immune response can be conceptualized as shifting from an early predominantly pro-inflammatory phase (left panel, red) to a later more pro-resolving phase (right panel, green), although the timing, magnitude, and subset specificity of this transition remain incompletely defined. (Left) Early phase (approximately 0–3 days): danger signals and CD1d-mediated antigen presentation are proposed to favor type I iNKT-cell activation, with associated IFN-γ, TNF-α, and IL-17 production. These responses are broadly consistent with M1-like microglial polarization, blood-brain barrier disruption, and secondary neuronal injury. Therapeutic approaches proposed for this window primarily aim to limit early inflammatory amplification, for example through transient CD1d blockade or S1P receptor modulation. Genetic models such as Cd1d−/− or Traj18−/− mice are better viewed as mechanistic proof-of-concept tools rather than therapeutic strategies. (Right) Later/subacute phase (approximately 3–7 days and beyond): NKT-associated responses may become increasingly linked to regulatory and reparative programs, including IL-4-, IL-10-, IL-13-, and TGF-β-associated pathways, with potential effects on pro-resolving myeloid polarization and tissue repair. Therapeutic approaches proposed for this stage include delayed or tolerogenic α-GalCer-based strategies, PPAR-γ agonists, or other immunomodulatory agents that may support endogenous resolution pathways. This schematic is intended as a working model rather than a definitive *in vivo* sequence.

If the proposed phase-dependent and context-dependent model of NKT-cell function in ICH is broadly correct, therapeutic strategies will likely need to move beyond simple global depletion or indiscriminate activation. Instead, the longer-term aim would be more precise immunomodulation: ideally, phase-aware and subset-informed intervention that attenuates early pro-inflammatory injury while preserving or enhancing later reparative programs. At present, however, most NKT-directed approaches remain preclinical or conceptual, and direct validation in ICH is limited. Existing strategies can be grouped into four broad categories: (1) direct modulation of iNKT cells using glycolipid ligands such as α-GalCer; (2) antibody-based interference with the CD1d-lipid-TCR axis; (3) genetic ablation models used for mechanistic proof-of-concept; and (4) indirect modulation through broader immunoregulatory agents, including S1P receptor modulators and PPAR-γ agonists, that may reshape the NKT activation milieu (22, 25, 30, 32, 33, 45, 48, 99, 125–128) (**Figure 5**).

### 5.1 Regulation by α-GalCer

α-GalCer, the prototypical synthetic glycolipid ligand for type I iNKT cells, is a useful tool for probing the therapeutic plasticity of NKT responses. In the setting of acute CNS injury, however, its effects are highly context dependent and appear to vary according to timing, dose, route, and the surrounding inflammatory milieu. As such, α-GalCer should currently be viewed primarily as a mechanistic probe and a conceptual therapeutic platform rather than an established treatment strategy for ICH.

#### 5.1.1 Timing may influence outcomes: an “immunomodulatory window”

The timing of α-GalCer administration is likely a major determinant of its immunological effect. Ultra-early activation (≤24 h) after ICH may amplify pathogenic iNKT-associated responses, including IFN-γ- and TNF-α-related signaling, microglial activation, and blood-brain barrier dysfunction (4, 7, 8, 11, 15, 18, 29, 34, 36, 72–75, 99, 129). Mechanistic studies further suggest that, in an inflamed microenvironment, strong TCR stimulation may bias iNKT cells toward NKT1- or NKT17-like phenotypes, thereby aggravating secondary brain injury (23, 24, 30, 50, 79, 99, 103, 105, 116).

In contrast, delayed or lower-dose administration (≥3 days) has been associated in some experimental systems with increased IL-4 and IL-10 production, consistent with NKT2- or NKT10-like programs (67–71, 79, 99, 110). A 2024 study in an LPS-induced neuroinflammation model reported that delayed α-GalCer treatment promoted IL-10-secreting NKT10-like responses and improved behavioral outcomes (130). However, direct evidence defining an optimal treatment window in ICH remains limited, and extrapolation from non-ICH models should be made cautiously.

Translational insight: These observations argue against indiscriminate early administration and instead support careful definition of a biomarker-informed therapeutic window. At present, α-GalCer should not be viewed as an immediately translatable intervention for acute ICH, but rather as a tool for exploring whether delayed, context-restricted NKT modulation can be achieved safely.

#### 5.1.2 Route and formulation: controlling where, and to what extent, α-GalCer acts

Systemic intravenous delivery of α-GalCer may lead to widespread iNKT activation, particularly in peripheral organs such as the liver, with consequent systemic cytokine release and, in some settings, induction of iNKT hyporesponsiveness or hepatotoxicity (23, 24, 32, 44, 45, 47, 49, 56, 99, 130). To mitigate these limitations, several delivery platforms have been proposed to improve spatial control and reduce systemic exposure:

1. Nanocarrier encapsulation: Nanocarrier and engineering-based strategies: Carrier-based formulations incorporating α-GalCer, including nanoparticle or lipid-based platforms, may alter biodistribution and tune downstream iNKT-cell activation in non-CNS settings, whereas engineered NKT-cell approaches highlight the broader translational potential of this axis; however, direct evidence for improved CNS delivery in ICH remains limited (66, 117, 131). In principle, such platforms may protect the glycolipid from degradation and reduce systemic exposure, although direct evidence for selective perihematomal targeting in ICH remains limited.
2. Intranasal delivery: This non-invasive route may provide partial nose-to-brain access and could reduce systemic exposure relative to intravenous delivery (132). However, its reproducibility, pharmacokinetics, and effective brain distribution in ICH remain uncertain.
3. DC-targeting liposomes: Liposomes decorated with ligands for dendritic-cell-associated receptors have been explored as a way to bias antigen presentation toward selected APC populations (132, 133). While conceptually attractive, this approach remains experimental and has not been validated in ICH.

Translational insight: Among these approaches, nanoparticle-based and intranasal delivery are conceptually attractive because they may improve CNS selectivity while reducing systemic exposure. However, their translational feasibility in ICH remains uncertain and will depend on reproducible manufacturing, biodistribution control, and patient-to-patient variability in BBB and nasal transport.

#### 5.1.3 Presenting α-GalCer in a tolerogenic context

The context in which α-GalCer is presented is likely to shape the functional output of iNKT cells. In experimental systems, delivery of α-GalCer via tolerogenic dendritic cells (tDCs), or in the presence of anti-inflammatory cytokines such as IL-10 or TGF-β, has been associated with an increased IL-10/IFN-γ ratio and with expansion of regulatory NKT-like programs, including NKT10-like populations (32, 33, 78, 79, 107, 110, 111, 118–120, 134). These observations suggest a potential route by which iNKT activation might be redirected toward immune regulation rather than inflammatory amplification.

Translational insight: While α-GalCer presentation in a tolerogenic context offers useful mechanistic insight into how regulatory NKT programs might be induced, its practical application in acute ICH is currently constrained by manufacturing complexity, timing, cost, and limited feasibility in emergency settings. At present, this strategy is best regarded as a conceptual platform rather than a near-term therapeutic option.

### 5.2 Monoclonal antibody applications

Antibody-based strategies directed at the CD1d-lipid-TCR interface or the invariant iNKT TCR may offer a more selective alternative to broad immunosuppression. Conceptually, these approaches could help blunt early pathogenic NKT activation while avoiding some of the limitations of non-specific immune suppression (22, 23, 25–28, 99, 125, 128, 135, 136). However, most supporting evidence comes from experimental systems outside ICH or from mechanistic extrapolation, and their relevance to human ICH remains to be established.

#### 5.2.1 Blocking antigen presentation (anti-CD1d)

Anti-CD1d antibodies function by hindering the loading and presentation of lipid antigens, thereby limiting activation of CD1d-restricted T cells, including both type I and type II NKT subsets (137, 138).

Proof of concept: In models of neurodegeneration and autoimmune neuroinflammation, CD1d blockade has been reported to dampen immune activation and reduce tissue injury, supporting the general concept that interruption of this axis can attenuate selected inflammatory cascades (11, 29, 34, 139, 140). Structural studies also indicate that some antibodies can distinguish among different lipid-bound states of CD1d, suggesting that modulation of antigen presentation may be tunable rather than purely binary (22, 23, 25, 28, 137, 138, 140).

Relevance to ICH: Direct ICH data remain sparse. Nevertheless, findings from other neuroinflammatory settings support the hypothesis that interrupting CD1d-dependent antigen presentation might help limit early cytokine amplification and perihematomal edema. A major caveat is that CD1d blockade is not subset selective and would affect both potentially pathogenic and potentially reparative NKT-associated programs. Accordingly, any such strategy would likely require careful temporal restriction and should currently be viewed as experimental.

#### 5.2.2 Selective inhibition or depletion of type I iNKT cells (anti-iTCR)

A more selective approach is to target the semi-invariant TCR α chain expressed by type I iNKT cells.

Representative agents: Representative agents: The murine NKT14 antibody binds specifically to the invariant iNKT TCR. In murine systems, Fc engineering enables either depletion (NKT14) or activation (NKT14m) of iNKT cells (101). Compared with older anti-Vβ8 reagents, they offer greater relative specificity for type I iNKT cells (10, 99, 125, 128).

Conceptual rationale: This approach is motivated by the working hypothesis that type I iNKT-associated responses may contribute more prominently to early inflammatory injury, whereas some non-type-I NKT programs may be relatively more regulatory. In principle, selective invariant TCR targeting could therefore bias the overall NKT-associated immune landscape toward a less inflammatory state (24, 30, 46, 51, 52, 55, 125, 128). In clinical stroke cohorts, elevated iNKT activation has been associated with poorer outcomes, providing an indirect rationale for further investigation (11, 29, 34–36, 42, 43, 141, 142).

Critical appraisal: Compared with global CD1d blockade, invariant TCR-targeted strategies may offer a more selective conceptual approach in settings where type I iNKT responses are thought to dominate early injury. However, this rationale remains to be tested directly in ICH, and important translational barriers include biologic cost, immunogenicity, dosing control, and the possibility of impairing antimicrobial defense.

### 5.3 Gene knockout models

Genetic ablation models are not therapeutic strategies in themselves, but they remain essential for mechanistic dissection and for benchmarking pharmacological interventions. Their value lies in clarifying whether, when, and in which compartments NKT-cell pathways contribute to ICH pathology.

#### 5.3.1 CD1d−/− mice

CD1d is the obligate restriction element for CD1d-restricted lipid-reactive T cells. Mice with targeted disruption of the Cd1d gene are therefore deficient in both type I and type II NKT subsets (22–24, 30, 44–46). In the absence of these cells, animals fail to mount the rapid NKT-associated cytokine responses characteristic of wild-type mice.

Relevance to ICH: Direct evidence in ICH remains limited. However, studies in ischemic stroke and experimental autoimmune neuroinflammation suggest that global NKT deficiency can attenuate early neuroinflammation and reduce tissue injury (11, 18, 29, 34, 37, 140, 141). CD1d−/− mice therefore provide a useful “global loss-of-function” framework for testing whether aggregate NKT-associated responses contribute to acute perihematomal injury. A major caveat is the simultaneous loss of potentially reparative type II or regulatory NKT-associated programs, which complicates interpretation of later recovery-phase findings.

#### 5.3.2 Traj18−/− mice

Type I iNKT cells are defined by their invariant TCR α chain involving the Traj18 gene segment.

Methodological note: Early Jα18-deficient strains disrupted additional upstream TCRα segments and inadvertently narrowed the overall TCR repertoire (55). Newer Traj18−/− lines generated by more precise genome-editing strategies preserve broader TCR diversity and are therefore better suited for mechanistic work (55, 103, 106).

Relevance to ICH: These refined models are useful for isolating the contribution of type I iNKT cells, which are often hypothesized to be important in early inflammatory responses after acute CNS injury (11, 18, 29, 34, 129, 142). Comparison of Traj18−/− and CD1d−/− mice may, in principle, help infer the relative roles of type I versus non-type-I NKT-associated programs. However, such inference remains indirect and depends heavily on model design, timing, and the specific knockout strain used.

#### 5.3.3 Conditional knockout strategies

To move beyond global deletions and better dissect where and when NKT-CD1d interactions may be relevant, conditional knockout strategies could be particularly informative.

Cell-type-specific targeting: Crossing Cd1d-floxed mice with myeloid or microglia-associated Cre drivers may allow investigators to assess whether local antigen presentation by brain-resident or infiltrating myeloid populations is sufficient to support NKT-associated inflammatory programs (107, 124, 125).

Temporal control: Inducible Cre-ERT2 systems could, in principle, permit deletion of Cd1d during selected phases of disease, for example during the acute phase versus later recovery (124, 125). Combined with higher-dimensional readouts such as single-cell profiling and spatial approaches, such models may help define when and where NKT engagement is most relevant after ICH (4, 5, 18, 19, 72–74).

Although technically demanding, these conditional approaches may provide a more informative framework for identifying the cellular interactions and temporal windows most relevant for future therapeutic design.

### 5.4 Drug intervention

Broader immunomodulatory agents may influence NKT-cell activation, trafficking, or effector polarization indirectly, even though they are not NKT specific. Their main translational advantage is that some already have human safety data in other indications, making them relevant from a drug-repurposing perspective. However, any benefit observed in ICH should not be assumed to reflect NKT modulation alone.

#### 5.4.1 Sphingosine-1-phosphate receptor modulators

Fingolimod (FTY720), a functional antagonist of S1P1 receptors, limits lymphocyte egress from secondary lymphoid organs and thereby reduces peripheral immune-cell trafficking (130, 143).

Proof of concept: In an early clinical study in ICH, fingolimod treatment was associated with reduced perihematomal edema and improved neurological recovery relative to standard care. Preclinical studies likewise suggest that S1P receptor modulation can limit lymphocyte trafficking, preserve BBB integrity, and attenuate secondary injury. Because NKT-cell migration is influenced by S1P gradients, one plausible interpretation is that fingolimod may also reduce recruitment or redistribution of pathogenic iNKT-associated responses during the acute phase. However, its effects are not NKT specific, and the relative contribution of NKT-cell sequestration to the observed benefit remains uncertain (11, 23, 24, 29, 30, 32, 34, 130, 132, 143, 144).

Critical appraisal: As one of the few immunomodulators with preliminary clinical data in ICH, fingolimod is an informative repurposing candidate. However, its non-selective mechanism and potential side effects, including bradycardia, lymphopenia, and infection risk, require caution. Any clinical benefit should not be interpreted as evidence of specific NKT-targeted blockade.

#### 5.4.2 PPAR-γ agonists

Peroxisome proliferator-activated receptor-γ *PPAR*–*γ* agonists, such as pioglitazone, regulate lipid metabolism and inflammatory signaling. Activation of PPAR-γ can suppress NF-κB-dependent inflammatory pathways and has been associated with increased IL-10-related signaling and a more resolution-favorable immune environment (133, 145).

Mechanistic link: In experimental ICH models, pioglitazone has been associated with PPAR-γ upregulation, activation of the Nrf2-GPx4 axis, inhibition of ferroptosis, and promotion of M2-like microglial polarization, together with improved functional outcomes (80, 82). Studies in other inflammatory settings further suggest that PPAR-γ activation may bias NKT-associated responses toward a more regulatory phenotype, including increased IL-10 production (79, 110, 111, 133, 145).

Critical appraisal: PPAR-γ agonists may therefore provide a potentially useful combination of neuroprotective and immunoregulatory effects. However, the extent to which these benefits in ICH are mediated specifically through NKT-cell reprogramming remains unresolved, and direct NKT-focused validation is still needed.

#### 4.4.3 Traditional Chinese medicine-derived compounds

Traditional Chinese medicine-derived compounds may provide a source of pleiotropic immunomodulators relevant to ICH. Agents such as celastrol or ligustrazine have been reported to influence inflammatory pathways associated with Th1/Th17 restraint or pro-resolving immune states (67, 68, 70, 134–136). However, direct evidence linking these compounds to NKT-specific modulation in ICH is currently limited. At present, their value is best regarded as hypothesis-generating, and future studies will need to incorporate NKT-resolved readouts to determine whether they truly influence NKT activation state, trafficking, or subset balance.

Overall, current therapeutic strategies for NKT modulation in ICH span a spectrum from highly specific approaches (e.g., monoclonal antibodies) to broader immunomodulatory agents (e.g., S1P modulators, PPAR-γ agonists, and TCM-derived compounds). To facilitate conceptual comparison, we have summarized representative agents, their mechanisms of action, key advantages, safety limitations, and current developmental stages in **Table 1** (see the end of the manuscript).

**Table 1:** Conceptual and experimental therapeutic strategies targeting NKT cells in intracerebral hemorrhage (ICH).

Strategy/class	Representative agents	Primary target/mechanism	NKT subset & ICH phase focus	Key advantages	Key caveats & development stage
α-GalCer agonism	α-GalCer (KRN7000); nanoparticles; intranasal α-GalCer	Activates type I iNKT; outcome depends on timing, dose, and route.	Early: NKT1/17 (pro-injury); Late: NKT2/10 (pro-resolving).	Strong immunomodulation; nanocarriers/intranasal delivery reduce systemic toxicity.	Timing-dependent (cytokine storm risk); significant BBB delivery challenges; lacks direct clinical validation; data remains inferential.
Anti-CD1d antibodies	CD1d-blocking monoclonal antibodies	Blocks CD1d lipid presentation and downstream TCR signaling.	Affects all NKT subsets (type I & II); targets early injury phase.	Upstream control of neuroinflammation; dampens IFN-γ/TNF-α cascades.	Non-selective (suppresses protective type II NKT); risk of broad immunosuppression (e.g., impaired antimicrobial defense); preclinical stage.
Anti-iTCR antibodies	NKT14/NKT14m (mouse); NKTT320 (humanized)	CD1d-blocking monoclonal antibodies	Blocks CD1d lipid presentation and downstream TCR signaling.	Affects all NKT subsets (type I & II); targets early injury phase.	Upstream control of neuroinflammation; dampens IFN-γ/TNF-α cascades.
S1P receptor modulators	Fingolimod (FTY720); siponimod	Antagonizes S1P_1_; traps lymphocytes in lymphoid organs, reducing CNS trafficking.	Restricts early NKT and T-cell brain influx; hyperacute/acute phase.	Existing clinical safety profile; proof-of-concept ICH trial shows reduced edema.	Broad lymphocyte suppression; infection and lymphopenia risks; requires optimized dose/duration for ICH.
PPAR-γ agonists	Pioglitazone; other PPAR-γ agonists	Nuclear receptor activation; promotes M2 macrophage and regulatory NKT phenotypes.	Subacute/reparative phase; favors anti-inflammatory IL-10 programs.	Broad neuroprotection; existing clinical safety data in metabolic diseases.	Systemic side effects (fluid retention, cardiovascular risks); direct NKT-specific ICH data remains limited.
TCM-derived compounds	Celastrol; ligustrazine; ginkgolide B	Multi-target anti-inflammatory and neuroprotective actions (e.g., IL-4/M2 bias).	Broadly targets early injury and late repair phases.	Mild toxicity profile; compatible with combination strategies.	Mechanistic link to NKT cells remains largely speculative; complex PK/PD profiles; requires rigorous target validation.

## 6 Challenges and future perspectives

Despite growing insight into NKT-cell biology, translating these observations into clinically useful therapeutic strategies for ICH remains challenging. Key obstacles include NKT-cell rarity, phenotypic heterogeneity, context-dependent functional plasticity, and incomplete understanding of how these cells interact with the neurovascular and systemic immune compartments over time. Importantly, much of the current framework is derived from preclinical studies, whereas direct human evidence defining NKT-cell kinetics, subset composition, and tissue localization after ICH remains limited. These issues complicate both mechanistic interpretation and clinical translation.

### 6.1 Current limitations

Technical scarcity and identification challenges. NKT cells, particularly type II and regulatory subsets, are rare and lack fully exclusive surface markers, which complicates their reliable isolation, quantification, and functional characterization in clinical samples. This scarcity limits routine immune monitoring and hinders development of robust biomarkers.

Limitations of the current evidence supporting the phase-dependent model. Although a phase-dependent framework is useful for integrating the available literature, the evidence supporting this model remains incomplete. First, much of the current evidence is derived from preclinical studies, whereas direct human data on NKT-cell kinetics, subset composition, and tissue localization after ICH are still sparse. Second, several mechanistic inferences are extrapolated from broader NKT biology or related CNS injury models rather than being demonstrated directly in ICH. Third, currently available studies differ substantially in species, sampling compartments, time points, phenotyping strategies, and definitions of NKT-cell subsets, making cross-study comparison difficult. Finally, direct *in vivo* evidence demonstrating subset-specific switching of NKT cells during ICH progression remains limited. Accordingly, the proposed phase-dependent model should be regarded as a working framework rather than a conclusively established sequence of events.

Interspecies discrepancies. A substantial translational gap exists between murine and human NKT biology. Murine iNKT cells are relatively abundant in the liver and spleen, whereas human iNKT cells are rarer and may exhibit different tissue distribution and activation thresholds. These differences require caution when extrapolating preclinical findings to patients and underscore the need for humanized models and ex vivo studies using human blood, cerebrospinal fluid, and, where feasible, brain-associated specimens.

Functional plasticity and context dependence. NKT cells are not static entities; their phenotypes and cytokine programs may shift according to the evolving inflammatory milieu. Interventions that broadly suppress or activate NKT cells therefore risk blunting beneficial programs while failing to adequately control harmful ones. This context dependence highlights the limitations of simplistic on/off therapeutic strategies.

### 6.2 Translational challenges

Therapeutic timing. Defining the optimal window for intervention remains a central translational challenge. An intervention that is beneficial during one phase of ICH may be ineffective or even harmful at another stage. Early suppression of inflammatory NKT-associated responses could reduce secondary injury, yet excessive or prolonged inhibition might impair antimicrobial defense or later reparative processes.

Precision targeting. Current tools remain insufficiently subset selective. Strategies such as anti-CD1d blockade may suppress broad NKT-associated responses without adequately distinguishing between potentially pathogenic type I iNKT programs and more regulatory or reparative NKT-associated pathways. Developing approaches that more selectively modulate phase-relevant subsets remains an important goal.

Safety and systemic immune effects. Systemic manipulation of NKT cells may carry risks beyond the injured brain, including altered host defense, infection susceptibility, and unintended immune effects in peripheral organs such as the liver and spleen. These concerns are particularly relevant in ICH, where post-stroke immune dysfunction and infectious complications already influence outcome.

Delivery barriers. Efficient and selective delivery to the injured brain remains difficult. Although BBB disruption after ICH may transiently increase permeability, this process is heterogeneous across time and anatomical regions. As a result, the pharmacokinetics, tissue distribution, and safety of NKT-directed agents require careful evaluation rather than assuming that CNS access will be straightforward.

Patient stratification and biomarker selection. Inter-individual variability in age, sex, genetics, baseline immune tone, hematoma characteristics, and comorbidities adds considerable noise to translational studies and clinical trials. Future studies should therefore adopt stratified designs and identify biomarker-guided criteria to determine which patients may be most likely to benefit from early suppression versus later pro-resolving modulation of NKT-associated programs.

Lack of clinical validation. At present, no NKT-targeted intervention has been clinically validated in ICH. This gap underscores the need for more realistic translational strategies that incorporate safety, timing, biomarker selection, and clinically relevant outcome measures from the earliest stages of preclinical development. Together, these challenges indicate that future clinical development will likely require biomarker-guided, temporally stratified, and safety-conscious trial designs rather than uniform intervention paradigms.

### 6.3 Future directions

A major priority for the field is to generate a more reliable and NKT-focused cellular atlas of ICH across time and tissue compartments. Although single-cell RNA sequencing and spatial transcriptomics are often proposed as transformative tools, their application to NKT cells in ICH faces important technical constraints. NKT cells are rare, may be disproportionately lost during tissue dissociation and processing, and are not always reliably resolved by transcriptomic clustering alone. This issue is particularly relevant in ICH, where access to acute perihematomal human tissue is limited, and longitudinal studies often rely on peripheral blood or cerebrospinal fluid rather than direct sampling of the injured brain. In addition, conventional single-cell workflows may not confidently distinguish type I, type II, and regulatory NKT-like populations unless they are complemented by CD1d-tetramer-based enrichment, surface-protein profiling, or TCR-aware approaches. Therefore, the absence of a strong NKT-cell signal in existing ICH datasets should be interpreted cautiously, as it may reflect technical under-detection rather than true biological insignificance.

For these reasons, future studies will likely require integrated multimodal strategies rather than reliance on any single platform. Promising approaches include combinations of scRNA-seq with scTCR-seq, CITE-seq, flow cytometry, and CD1d-tetramer-based enrichment, ideally applied longitudinally across blood, cerebrospinal fluid, spleen, and experimental brain tissue. Such designs would help determine whether the proposed temporal shift in NKT-cell function reflects true phenotypic conversion, selective expansion of distinct subsets, or changing anatomical redistribution over the course of ICH.

A second priority is the development of clinically useful biomarkers that can define when NKT-directed intervention might be appropriate. Peripheral NKT-cell frequency, activation markers, cytokine signatures, and compartment-specific immune profiles should be evaluated in well-phenotyped prospective cohorts and linked to edema evolution, hematoma resolution, infection risk, and long-term neurological outcomes.

A third priority is to test phase-aware therapeutic strategies in a more rigorous manner. Rather than assuming that NKT cells should simply be inhibited or activated, preclinical studies should directly compare early transient suppression with later pro-resolving modulation using subset-informed and time-resolved designs. These studies should also incorporate safety monitoring, systemic immune readouts, and delivery considerations that better reflect the realities of clinical translation.

Only after these foundations are established will more ambitious engineering approaches—such as bioengineered NKT-based platforms, antibody-guided delivery systems, or advanced nanocarriers—become meaningfully testable in ICH. In this sense, future progress will depend not only on mechanistic discovery but also on aligning experimental design with the biological and translational complexity of NKT-cell responses after hemorrhagic stroke.

## 7 Conclusion

NKT cells appear to play an important immunoregulatory role at the interface of innate and adaptive immunity in intracerebral hemorrhage (ICH). Available evidence supports a working model in which NKT-cell responses are functionally heterogeneous and phase dependent. Early type I iNKT-associated responses may contribute to secondary injury by amplifying neuroinflammation, promoting pro-inflammatory myeloid activation, impairing blood-brain barrier integrity, and being associated with neutrophil recruitment. In later phases, regulatory NKT-associated programs, including type II NKT- or NKT10-like responses, may become increasingly linked to inflammatory resolution, hematoma clearance, and tissue repair through IL-4-, IL-10-, and related pathways.

This functional heterogeneity presents both a challenge and a potential therapeutic opportunity. Rather than favoring indiscriminate activation or suppression of NKT cells, current evidence suggests that stage-specific and subset-informed immunomodulation may represent a more realistic translational strategy. In principle, early attenuation of pathogenic NKT-associated inflammatory programs, followed by later support of regulatory or reparative NKT responses, could better align intervention with the evolving biology of ICH. However, this framework remains provisional and requires stronger validation in ICH-specific systems, particularly in human studies.

Looking ahead, high-resolution single-cell multi-omics, spatial profiling, and refined experimental models will be essential for defining the heterogeneity, tissue distribution, and temporal dynamics of human NKT cells after ICH. Integrating these mechanistic insights with biomarker-guided clinical studies may help identify actionable therapeutic windows and clarify which NKT-associated pathways are most suitable for intervention. Overall, NKT-cell biology may provide a useful framework for the development of more precise immunomodulatory approaches in ICH, although substantial mechanistic and translational gaps remain to be addressed.

## Glossary

α-GalCer: alpha-galactosylceramide
APC: antigen-presenting cell
AQP4: aquaporin-4
Arg1: arginase 1
BBB: blood–brain barrier
CCL2: C-C motif chemokine ligand 2
CCR2: C-C motif chemokine receptor 2
CD: cluster of differentiation (e.g., CD1d, CD4, CD8, CD40, CD40L, CD69, CD206)
CNS: central nervous system
CTLA-4: cytotoxic T-lymphocyte-associated protein 4
CXCL16: C-X-C motif chemokine ligand 16
CXCR6: C-X-C motif chemokine receptor 6
DAMP: damage-associated molecular pattern
DC: dendritic cell
DC-SIGN: dendritic cell-specific intercellular adhesion molecule-3-grabbing non-integrin
DN: double-negative (CD4⁻CD8⁻)
Foxp3: forkhead box P3
FTY720: fingolimod
GM-CSF: granulocyte–macrophage colony-stimulating factor
GPx4: glutathione peroxidase 4
HMGB1: high-mobility group box 1
HPA (axis): hypothalamic–pituitary–adrenal (axis)
ICAM-1: intercellular adhesion molecule-1
ICH: intracerebral hemorrhage
IFN-γ: interferon γ
Ig: immunoglobulin
IL: interleukin
IL-4Rα: interleukin-4 receptor alpha
IL-10R: interleukin-10 receptor
iNKT: invariant natural killer T
JAK1: Janus kinase 1
LFA-1: lymphocyte function-associated antigen-1
LNP: lipid nanoparticle
LPC: lysophosphatidylcholine
LPS: lipopolysaccharide
mAb: monoclonal antibody
MHC: major histocompatibility complex
MMP-9: matrix metalloproteinase-9
mTOR: mechanistic target of rapamycin
NET: neutrophil extracellular trap
NF-κB: nuclear factor kappa B
NK: natural killer
NKT: natural killer T
NLRP3: NLR family pyrin domain containing 3
Nrf2: nuclear factor erythroid 2–related factor 2
PD-1: programmed cell death protein 1
PD-L1: programmed death-ligand 1
PPAR-γ: peroxisome proliferator-activated receptor-γ
RAGE: receptor for advanced glycation end products
t: retinoic acid receptor-related orphan receptor γ
ROS: reactive oxygen species
S1P: sphingosine-1-phosphate
scRNA-seq: single-cell RNA sequencing
STAT: signal transducer and activator of transcription (e.g., STAT1, STAT3, STAT6)
TALEN: transcription activator-like effector nuclease
TCM: Traditional Chinese Medicine
TCR: T-cell receptor
tDC: tolerogenic dendritic cell
Th: T helper (e.g., Th1, Th2, Th17)
TLR: Toll-like receptor
TNF-α: tumor necrosis factor alpha
Treg: regulatory T cell
TYK2: tyrosine kinase 2
VCAM-1: vascular cell adhesion molecule-1
VLA-4: very late antigen-4.
